# Healing in Precious Stones

**Published:** 1892-10

**Authors:** 


### HEALING IN PRECIOUS STONES.

The father of jewelry was Prometheus. When he was cut loose by Hercules from the chains that fastened him to Mount Caucasus, he made a ring out of one of the links of his fetters, and in the bezel of it he fixed a portion of the rock. According to Pliny, that was the first ring and the first stone. Hebrew tradition says that the tablets of Moses were of sapphire. In Hebrew the word sappir means the most beautiful. It symbolizes loyalty, justice, beauty, and nobility. The emerald is mentioned by St. John in his Apocalypse. An emerald of inestimable value ornamented the bezel of the ring of Polycrates, King of Samos. That monarch, having been all his life favored by fortune, determined to put his luck to a severe test. He threw the ring into the sea. The next day he went fishing. The record of that day's sport still remains unbroken. His majesty caught a fine fish, and in the inside of the fish he found his ring. That happened in the year 230 of the foundation of Rome, and the ring, considered as a talisman, was placed among the royal treasures of the Temple of Concord. Emeralds from India, Persia and Peru are the most valuable. According to their tints and their luster they are classed as Prosines, Neronianes and Domitianes. According to Suetonius, Nero used to look at the fighting gladiators in his emerald. The stone is the emblem of charity, hope, joy and abundance. It had the reputation of curing epilepsy by application and of being an all-around pain killer.

The diamond has always been regarded as the most precious stone. It resists the hardest bodies. The Pontiff Aaron wore a diamond of astonishing virtues. It became obscure, almost black, when the Hebrews were in a state of mortal sin. If the guilty deserved death it became red, but in the presence of innocence it came back to its original purity and brilliancy. Rues assures us that diamonds breed, and that a certain princess of the house of Luxemburg had two which had a family in the course of a reasonable time. The same interesting assertion is also made by Boethius. The diamond was reputed as a preserver against epidemics and poisons. It calms anger and foments conjugal love. The ancients called it "the stone of reconciliation." It symbolizes constancy, strength and innocence.

The name of the precious stone inserted in the ring of Gyges has not been handed down to us, but it is probable that it was the topaz whose wonders Philostrates recounts in the life of Apollonius. An attribute of the sun and of fire, the ancients called it the gold magnet, as it was credited with the power of attracting that metal, indicating its veins and discovering treasures. Helliodorus, in his story of Theagenes and Caricles, says that the topaz saves from fire all those who wear it, and that Caricles was preserved by a topaz from the fiery vengeance of Arsaces, Queen of Ethiopia. This stone was one of the first talismans that Theagenes possessed in Egypt. The topaz at present symbolizes Christian virtues, faith, justice, temperance, gentleness, clemency.

One of the rarest and most precious stones is the carbuncle, which is sometimes confounded with the ruby, from which it differs by the intensity of its fires, produced by an internal luster of gold, while under the purple of the ruby there only appear dottings of azure or lacquer. Ethiopia produced the most precious ancient carbuncles. The Chaldeans regarded this stone as a powerful talisman. Legend makes the eyes of dragons out of carbuncles. Garcias ab Horto, physician of one of the viceroys of India, speaks of carbuncles which he saw in the palace of that prince which were so extraordinary in their brilliancy that they seemed "like red hot coals in the midst of darkness." Louis Vertoman reports that the King of Pegu wore an enormous one, which at night appeared to be lighted up with sunbeams. The virtues of the carbuncle are resistance to fire, preservation of the eyes, promotion of pleasant dreams, creation of happy illusions and an antidote against impure air.

The ruby is valued highest when it contains the least azure. The largest ruby that history speaks of belonged to Elizabeth of Austria, the wife of Charles IX. It was almost as big as a hen's egg. The virtues attributed to rubies are to banish sadness, to repress luxury and to drive away annoying thoughts. At the same time it symbolizes cruelty, anger and carnage, as well as boldness and bravery. A change in its color announces a calamity, but when the trouble is over it regains its primitive luster. The amethyst, so called from the Greek amethustos, meaning "not drunk," was a favorite stone among the Roman ladies. Its principal virtue was to draw away the vapors of inebriety from the brain. It also drove away evil thoughts and attracted to its possessor the favors of princes.

The opal, fallen from its ancient splendor, is to-day called an unlucky stone, even by those who laugh at old superstitions ; but it once held a high rank among precious stones. The belief that it attracted misfortune was founded on a Russian legend which found its way into France. The Empress Eugenie had a horror of an opal. At sight of one in the Tuilleries she manifested terror. That had the effect of lowering the price of the stone.

The turquoise is considered as a talisman in Persia, its native soil. It preserves its possessor from accidents and insures constancy in affections. The value of the turquoise depends on its shade and its size, especially its thickness. Those classed as belonging to the old rock are valued very highly. Emblem of youth, of sentiment and tender recollections, the turquoise may be called forget-me-not of stones. It breaks on the death of its proprietor and changes color when he is ill. This last observation is perfectly true and is certified to by all lapidaries. The same thing has been remarked of coral. "No only do precious stones live," says Jerome Cardan, "but they are liable to get sick, to suffer from the infirmities of old age and at last to die."

The most precious of all stones is the jade, on account of its rarity, its extraordinary qualities and the mystery of its cutting. It was regarded as a sacred stone and nobody had a right to possess it except a prince of imperial blood. Agerius Clutius, a famous physician in Amsterdam at the time of the Renaissance, published a work on the jade or nephritic stone, as it was then called on account of its action on the renal system. At the same period Italian authors spoke of the jade as osiada and discussed its wonderful powers for healing sciatica. The legends surrounding this stone abound in history. Good specimens of jade are extremely rare, and the world is at a loss to know how the Chinese managed to cut it, because it is so extremely hard that nothing can make an impression upon it. Splendid specimens of gray and green jade can be seen in the museum of the Trocadero.

---

Remedy for a Cold.—Bathe the legs in warm water at night, and take going to bed a drink of hot whey, with 4 grains of nitre. Cover warmly, so as to produce perspiration. If a sore throat, tie round it two or three folds of flannel sprinkled with spirits.

